# Isoquinolinium 5-(2,4-di­nitro­phen­yl)-1,3-dimethyl-2,6-dioxo-1,2,3,6-tetra­hydro­pyrimidin-4-olate: crystal structure, Hirshfeld surface analysis and pharmacological evaluation

**DOI:** 10.1107/S2056989016005004

**Published:** 2016-03-31

**Authors:** Ponnusamy Poornima Devi, Doraisamyraja Kalaivani

**Affiliations:** a PG and Research Department of Chemistry, Seethalakshmi Ramaswami College, Tiruchirappalli 620 002, Tamil Nadu, India

**Keywords:** crystal structure, 3D Hirshfeld analysis, 1,3-di­methyl­barbituric acid, anti­convulsant activity, hypnotic activity

## Abstract

The asymmetric unit of the title salt C_9_H_8_N^+^·C_12_H_9_N_4_O_7_^−^, which exhibits anti­convulsant and hypnotic activities, comprises one anion and one cation inter­acting *via* an N—H⋯O hydrogen bond. In the anion, the six-membered rings are inclined each to other at 42.78 (9)°. The nitro groups in the 2,4-di­nitro­phenyl fragment attached to the aromatic ring in the *para* and *ortho* positions are twisted from its plane by 3.1 (2) and 45.5 (2)°, respectively. In the crystal, weak C—H⋯O hydrogen bonds consolidate the crystal packing. The Hirshfeld surface analysis revealed that O⋯H/H⋯O inter­molecular contacts predominate in the crystal packing.

## Abstract 2

The title compound, which comprises an isoquinolinium cation and 5-(2,4-di­nitro­phen­yl)-1,3-di­methyl­babriturate anion, exhibits anti­convulsant and hypnotic activities. 3D Hirshfeld surface analysis establishes the predominant O⋯H/H⋯O inter­molecular contacts in the crystal lattice.

## Chemical context   

Barbiturates play a significant role in biological systems (Hueso-Ureña *et al.*, 2003 ▸). Epilepsy (convulsion) is a life-threatening neurological disorder which requires immediate treatment with suitable drugs (Shorvon, 2004 ▸). Barbiturates have been proved to be potent drugs for this dreadful disorder (Nadkarni *et al.*, 2005 ▸). The iso­quinoline unit also displays a wide spectrum of activity and it is an important component of many biologically active alkaloids (Montalban, 2011 ▸). Since 2008, we have been periodically synthesizing new barbiturate derivatives and exploring their anti­convulsant activity (Kalaivani *et al.*, 2008 ▸; Kalaivani & Malarvizhi, 2009 ▸; Kalaivani & Buvaneswari, 2010 ▸; Manickkam & Kalaivani, 2011 ▸; Babykala & Kalaivani, 2012 ▸; Buvaneswari & Kalaivani, 2013 ▸; Vaduganathan & Doraisamyraja, 2014 ▸; Gomathi & Kalaivani, 2015 ▸). The title mol­ecular salt, which is a new derivative of 1,3-di­methyl­barbituric acid (barbiturate), was recently obtained by our group. Herewith we report its crystal structure.
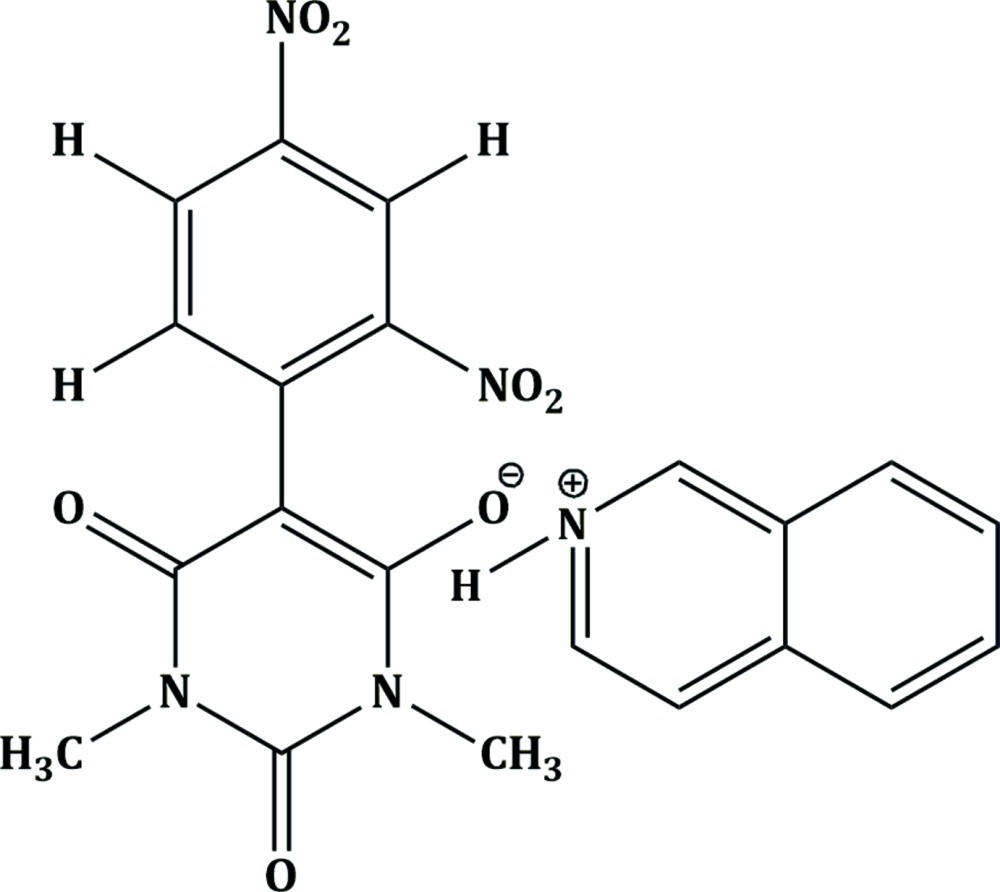


## Structural commentary   

**Figure 1:**
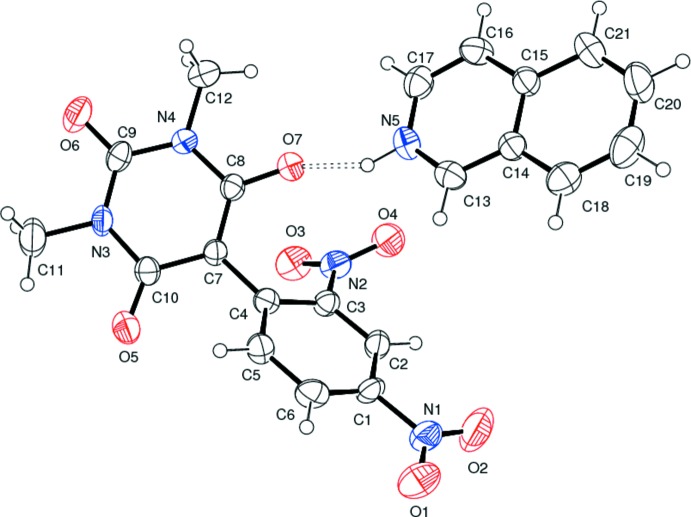
The asymmetric unit of (I) showing the atom numbering and 40% probability displacement ellipsoids. The doubled-dashed line denotes the N—H⋯O hydrogen bond between the cation and anion.

In the title compound, (I) (Fig. 1 ▸), all the bond lengths and bond angles are normal and comparable with those observed in the related barbiturates (Sridevi & Kalaivani, 2012 ▸; Gunaseelan & Doraisamyraja, 2014 ▸). The plane of the di­nitro­aromatic ring C1–C6 and that of the barbiturate ring C7/C8/N4/C9/N3/C10 form a dihedral angle of 42.78 (9)°. The nitro groups in the 2,4-di­nitro­phenyl fragment attached to the aromatic ring in the *para* and *ortho* positions are twisted from its plane by 3.1 (2) and 45.5 (2)°, respectively. Thus the *para* nitro group is more involved in delocalizing the negative charge than the *ortho* nitro group in the anionic part. This sort of delocalization of the charge over a large area imparts a maroon red colour to the title compound.

## Supra­molecular features   

**Figure 2:**
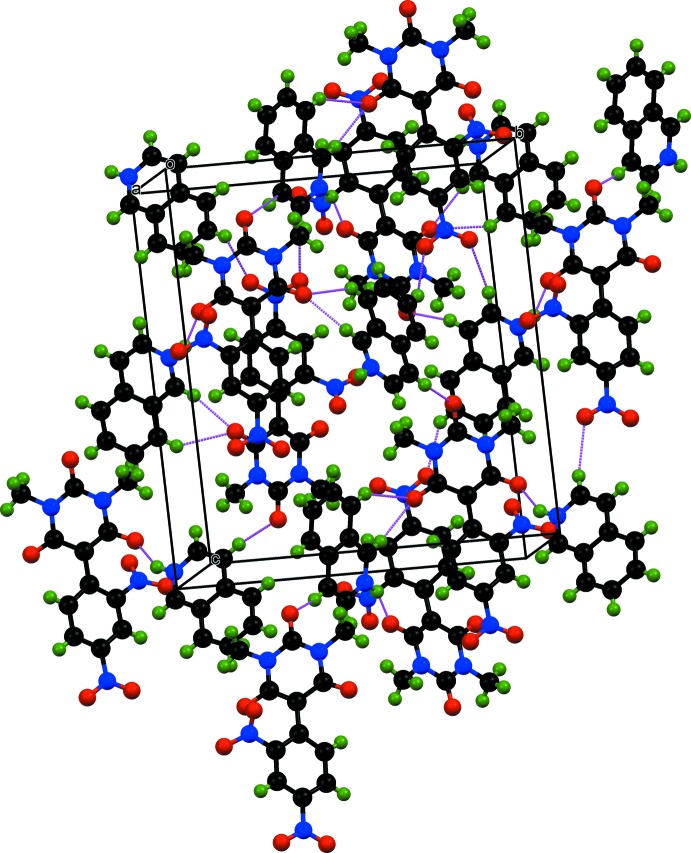
Crystal packing of (I) viewed approximately down the *a* axis. Hydrogen bonds are shown as purple dotted lines.

The aminium group is involved in formation of an N—H⋯O hydrogen bond (Table 1 ▸) between the isoquinolinium cation (N5—H5*A*) and the deprotonated enol oxygen atom O7. In the crystal, weak C—H⋯O hydrogen bonds (Table 1 ▸) consolidate the crystal packing (Fig. 2 ▸). An 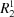
(6) motif is generated by the C—H groups [C13—H13 and C20—H20] of the isoquinolinium cation and oxygen atom O5 of the carbonyl group of the barbiturate ring of the anion. Although there are three rings with cyclically delocalized π electron clouds, no π–π stacking inter­actions are observed between them.

## 3D Hirshfeld Surface Analysis and 2D Fingerprint Analysis   

**Figure 3:**
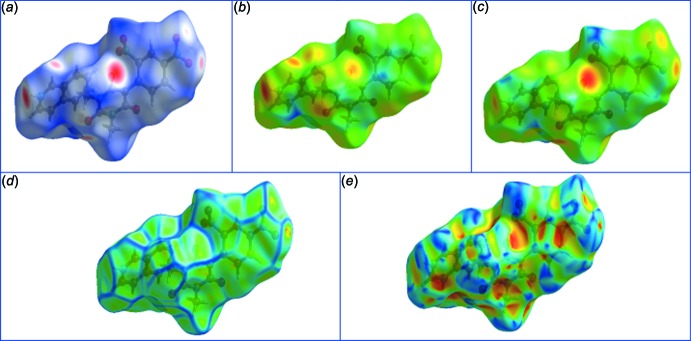
3D Hirshfeld surface analysis of (I) mapped over (*a*) *d*_norm_ ranging from −0.723 (red) to 1.464 (blue); (*b*) *d*_e_; (*c*) *d*_i_; (*d*) curvedness; (*e*) shapeindex.

**Figure 4:**
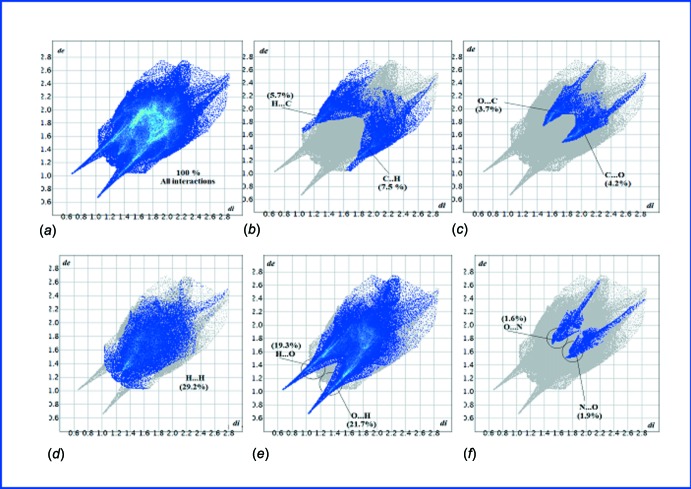
2D Fingerprint plots showing contributions from different contacts: (*a*) overall inter­actions; (*b*) C⋯H/H⋯C; (*c*) C⋯O/O⋯C; (*d*) H⋯H; (*e*) O⋯H/H⋯O and (*f*) N⋯O/O⋯N.

**Figure 5:**
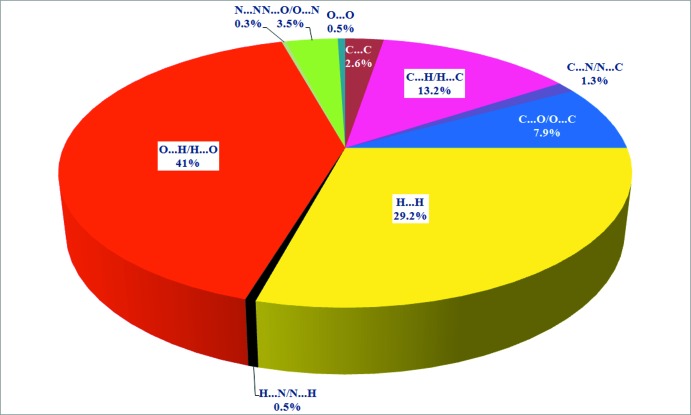
Pie chart showing the qu­anti­tative distribution of inter­molecular inter­actions in (I).

Hirshfeld surfaces (Spackman & Jayatilaka, 2009 ▸) and the associated 2D-fingerprint plots (McKinnon *et al.*, 2007 ▸) of the title mol­ecular salt have been generated using *Crystal Explorer 3.1* (Wolff *et al.*, 2013 ▸). Hirshfeld surfaces mapped with different properties, *e.g. d*_e_, *d*_norm_, *d*_i_, shapeindex, curvedness, have proven to be a useful visualization tool for the analysis of inter­molecular inter­actions. The 2D-fingerprint plots of Hirshfeld surfaces have been used to pinpoint and scrutinize the percentage of hydrogen-bonding inter­actions present in the crystal structure. The presented graphical plots use the same red-white-blue color scheme, wherein red highlights the shortest inter­molecular atomic contacts (negative *d*_norm_ values), white is used for contacts around the van der Waals separation, and blue corresponds to longer ones (positive *d*_norm_ values). Hirshfeld surface analysis of the new barbiturate of present inter­est has *d*_norm_ values ranging from −0.723 (red) to 1.464 (blue), as specified in Fig. 3 ▸. The globularity value (a measure of the degree to which the surface area differs from that of the shape) is less than 1 (0.743), implying a more structured mol­ecular surface and it is an oblate object (asphericity, 0.282). 2D-Fingerprint plots showing contributions from different contacts: (*a*) overall inter­actions (*b*) C⋯H/H⋯C (*c*) C⋯O/O⋯C (*d*) H⋯H (*e*) O⋯H/H⋯O (*f*) N⋯O/O⋯N are depicted in Fig. 4 ▸, and Fig. 5 ▸ (pie chart) clearly demonstrates that the O⋯H/H⋯O inter­actions dominate in the crystal.

## Pharmacological activity   

Epilepsy affects about 0.5% of the world’s population. A seizure is caused by an asynchronous high-frequency discharge of a group of neurons, starting locally and spreading to a varying extent to affect other parts of the brain. 1,3-Di­methyl­barbituric acid is the most significant compound with a heterocyclic structure and exists in two tautomeric forms (keto and enol) due to the mobility of active methyl­ene group hydrogen atoms in its mol­ecule. Barbiturates are drugs that act as central nervous system depressants and can therefore produce a wide spectrum of effects from mild sedation to total anaesthesia. They are also effective as anxiolytics, hypnotics and anti­convulsants. As the mol­ecular salt of the present investigation is a derivative of 1,3-di­methyl­barbituric acid, it has been subjected to the Maximal Electro Shock method to evaluate its anti­convulsant activity (Misra *et al.*,1973 ▸; Kulkarni, 1999 ▸). It reduces all phases of convulsion (tonic-flexor, tonic-extensor, clonic-convulsion and stupor) even at low dosage (25 mg kg^−1^) and the animals recovered after the experiment.

## Synthesis and crystallization   

1-Chloro-2,4-di­nitro­benzene (2.02 g, 0.01 mol) in 40 mL of absolute alcohol was mixed with 1,3-di­methyl­barbituric acid (1.56 g, 0.01 mol) in 30 mL ethanol. To this mixture, 0.02 mol of iso­quinoline was added and the mixture was shaken well for 5 h and kept as such for 24 h. Excess ethanol was removed through evaporation. A maroon-red pasty mass was obtained. This paste was digested with hot ethanol to obtain a maroon-red solid. The solid deposited at the bottom of the flask was filtered, powdered well using an agate mortar, washed again with 20 mL of dry ether and recrystallized from absolute alcohol. Good quality single crystals suitable for X-ray diffraction analysis were obtained by slow evaporation of ethanol at room temperature (yield: 80%; m.p. 413 K).

## Refinement   

Crystal data, data collection and structure refinement details are summarized in Table 2 ▸. The N-bound H atom was located in a difference Fourier map and refined isotropically. C-bound H atoms were positioned geometrically and refined as riding, with C—H = 0.93–0.96 Å and *U*_iso_(H) = 1.2–1.5 *U*_eq_(C).

## Supplementary Material

Crystal structure: contains datablock(s) global, I. DOI: 10.1107/S2056989016005004/cv5504sup1.cif

Structure factors: contains datablock(s) I. DOI: 10.1107/S2056989016005004/cv5504Isup2.hkl

Supporting information file. DOI: 10.1107/S2056989016005004/cv5504Isup3.cml

CCDC reference: 1444879

Additional supporting information:  crystallographic information; 3D view; checkCIF report

## Figures and Tables

**Table 1:** Hydrogen-bond geometry (Å, °)

*D*—H⋯*A*	*D*—H	H⋯*A*	*D*⋯*A*	*D*—H⋯*A*
N5—H5*A*⋯O7	0.93 (6)	1.74 (6)	2.592 (6)	150 (5)
C13—H13⋯O5^i^	0.93	2.40	3.260 (7)	153
C16—H16⋯O6^ii^	0.93	2.33	3.187 (7)	154
C17—H17⋯O2^iii^	0.93	2.61	3.424 (8)	146

Symmetry codes: (i) 
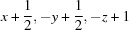
; (ii) 
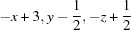
; (iii) 
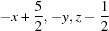
.

**Table 2:** Experimental details

Crystal data	
Chemical formula	C_9_H_8_N^+^·C_12_H_9_N_4_O_7_^−^
*M*_r_	451.40
Crystal system, space group	Orthorhombic, *P*2_1_2_1_2_1_
Temperature (K)	296
*a*, *b*, *c* (Å)	7.5315 (3), 15.5640 (8), 17.3901 (8)
*V* (Å^3^)	2038.47 (16)
*Z*	4
Radiation type	Mo *K*α
μ (mm^−1^)	0.11
Crystal size (mm)	0.35 × 0.30 × 0.25

Data collection	
Diffractometer	Bruker Kappa APEXII CCD
Absorption correction	Multi-scan (*SADABS*; Bruker, 2004 ▸)
*T*_min_, *T*_max_	0.958, 0.984
No. of measured, independent and observed [*I* > 2σ(*I*)] reflections	27633, 3588, 2797
*R*_int_	0.038
(sin θ/λ)_max_ (Å^−1^)	0.594

Refinement	
*R*[*F* ^2^ > 2σ(*F* ^2^)], *wR*(*F* ^2^), *S*	0.055, 0.164, 1.11
No. of reflections	3588
No. of parameters	302
H-atom treatment	H atoms treated by a mixture of independent and constrained refinement
Δρ_max_, Δρ_min_ (e Å^−3^)	0.29, −0.26
Absolute structure	Flack *x* determined using 1033 quotients [(*I* ^+^)−(*I* ^−^)]/[(*I* ^+^)+(*I* ^−^)] (Parsons *et al.*, 2013 ▸)
Absolute structure parameter	0.5 (4)

Computer programs: *APEX2*, *SAINT* and *XPREP* (Bruker, 2004 ▸), *SIR92* (Altomare *et al.*, 1993 ▸), *SHELXL2014* (Sheldrick, 2015 ▸), *ORTEP-3 for Windows* (Farrugia, 2012 ▸) and *Mercury* (Macrae *et al.*, 2008 ▸).

## supplementary crystallographic information

### Crystal data

**Table:**

C_9_H_8_N^+^·C_12_H_9_N_4_O_7_^−^	*D*_x_ = 1.471 Mg m^−^^3^
*M**_r_* = 451.40	Mo *K*α radiation, λ = 0.71073 Å
Orthorhombic, *P*2_1_2_1_2_1_	Cell parameters from 9400 reflections
*a* = 7.5315 (3) Å	θ = 2.3–24.0°
*b* = 15.5640 (8) Å	µ = 0.11 mm^−^^1^
*c* = 17.3901 (8) Å	*T* = 296 K
*V* = 2038.47 (16) Å^3^	Block, brown
*Z* = 4	0.35 × 0.30 × 0.25 mm
*F*(000) = 936	

### Data collection

**Table:**

Bruker Kappa APEXII CCD diffractometer	3588 independent reflections
Radiation source: fine-focus sealed tube	2797 reflections with *I* > 2σ(*I*)
Graphite monochromator	*R*_int_ = 0.038
ω and φ scan	θ_max_ = 25.0°, θ_min_ = 2.3°
Absorption correction: multi-scan (*SADABS*; Bruker, 2004)	*h* = −8→8
*T*_min_ = 0.958, *T*_max_ = 0.984	*k* = −18→18
27633 measured reflections	*l* = −20→20

### Refinement

**Table:**

Refinement on *F*^2^	Hydrogen site location: mixed
Least-squares matrix: full	H atoms treated by a mixture of independent and constrained refinement
*R*[*F*^2^ > 2σ(*F*^2^)] = 0.055	*w* = 1/[σ^2^(*F*_o_^2^) + (0.0596*P*)^2^ + 2.2423*P*] where *P* = (*F*_o_^2^ + 2*F*_c_^2^)/3
*wR*(*F*^2^) = 0.164	(Δ/σ)_max_ < 0.001
*S* = 1.11	Δρ_max_ = 0.29 e Å^−^^3^
3588 reflections	Δρ_min_ = −0.26 e Å^−^^3^
302 parameters	Absolute structure: Flack *x* determined using 1033 quotients [(*I*^+^)-(*I*^-^)]/[(*I*^+^)+(*I*^-^)] (Parsons *et al.*, 2013)
0 restraints	Absolute structure parameter: 0.5 (4)

### Special details

**Table:**

Geometry. All esds (except the esd in the dihedral angle between two l.s. planes) are estimated using the full covariance matrix. The cell esds are taken into account individually in the estimation of esds in distances, angles and torsion angles; correlations between esds in cell parameters are only used when they are defined by crystal symmetry. An approximate (isotropic) treatment of cell esds is used for estimating esds involving l.s. planes.
Refinement. The elements in the sample do not have sufficient anomalous scattering power for Mo(kα) radiation. Hence the Flack parameter and its standard deviation obtained from refinement have no physical significance.

### Fractional atomic coordinates and isotropic or equivalent isotropic displacement parameters (Å^2^)

**Table:**

	*x*	*y*	*z*	*U*_iso_*/*U*_eq_	
C1	1.0128 (8)	0.1849 (4)	0.6047 (3)	0.0420 (13)	
C2	0.9775 (8)	0.1140 (3)	0.5603 (3)	0.0430 (13)	
H2	0.9489	0.0617	0.5830	0.052*	
C3	0.9850 (7)	0.1217 (3)	0.4818 (3)	0.0380 (12)	
C4	1.0322 (7)	0.1979 (3)	0.4434 (3)	0.0360 (11)	
C5	1.0660 (8)	0.2679 (3)	0.4922 (3)	0.0443 (13)	
H5	1.0970	0.3204	0.4705	0.053*	
C6	1.0549 (8)	0.2615 (4)	0.5715 (3)	0.0495 (14)	
H6	1.0761	0.3094	0.6021	0.059*	
C7	1.0594 (7)	0.2047 (3)	0.3605 (3)	0.0337 (11)	
C8	1.1510 (7)	0.1402 (3)	0.3218 (3)	0.0357 (11)	
C9	1.1333 (8)	0.2196 (3)	0.2030 (3)	0.0421 (13)	
C10	1.0069 (7)	0.2807 (3)	0.3207 (3)	0.0388 (12)	
C11	0.9855 (12)	0.3574 (4)	0.1982 (4)	0.072 (2)	
H11A	0.9241	0.3963	0.2317	0.108*	
H11B	1.0872	0.3856	0.1766	0.108*	
H11C	0.9074	0.3396	0.1576	0.108*	
C12	1.2891 (9)	0.0820 (4)	0.2065 (3)	0.0569 (16)	
H12A	1.3142	0.0369	0.2426	0.085*	
H12B	1.2236	0.0590	0.1639	0.085*	
H12C	1.3985	0.1062	0.1884	0.085*	
C13	1.4554 (8)	0.0032 (4)	0.5236 (3)	0.0486 (14)	
H13	1.4114	0.0499	0.5511	0.058*	
C14	1.5694 (7)	−0.0536 (3)	0.5585 (3)	0.0404 (12)	
C15	1.6326 (7)	−0.1244 (4)	0.5152 (3)	0.0449 (13)	
C16	1.5786 (8)	−0.1322 (4)	0.4383 (3)	0.0522 (15)	
H16	1.6195	−0.1776	0.4084	0.063*	
C17	1.4675 (8)	−0.0738 (4)	0.4083 (3)	0.0546 (15)	
H17	1.4313	−0.0788	0.3573	0.065*	
C18	1.6259 (8)	−0.0443 (4)	0.6357 (3)	0.0554 (15)	
H18	1.5888	0.0026	0.6647	0.066*	
C19	1.7348 (9)	−0.1043 (6)	0.6669 (4)	0.069 (2)	
H19	1.7689	−0.0991	0.7181	0.083*	
C20	1.7960 (10)	−0.1728 (5)	0.6242 (4)	0.074 (2)	
H20	1.8716	−0.2126	0.6471	0.088*	
C21	1.7487 (9)	−0.1835 (4)	0.5502 (4)	0.0642 (18)	
H21	1.7926	−0.2298	0.5223	0.077*	
N1	1.0035 (8)	0.1760 (4)	0.6875 (3)	0.0628 (15)	
N2	0.9243 (7)	0.0456 (3)	0.4385 (3)	0.0496 (12)	
N3	1.0427 (7)	0.2826 (3)	0.2415 (2)	0.0435 (11)	
N4	1.1845 (6)	0.1485 (3)	0.2441 (2)	0.0389 (10)	
N5	1.4081 (7)	−0.0079 (3)	0.4514 (3)	0.0508 (13)	
O1	1.0316 (9)	0.2406 (4)	0.7261 (3)	0.0887 (18)	
O2	0.9721 (10)	0.1063 (4)	0.7153 (3)	0.099 (2)	
O3	0.8197 (6)	0.0553 (3)	0.3865 (3)	0.0632 (12)	
O4	0.9781 (7)	−0.0246 (3)	0.4618 (3)	0.0690 (14)	
O5	0.9298 (6)	0.3428 (3)	0.3499 (2)	0.0578 (11)	
O6	1.1665 (7)	0.2254 (3)	0.1336 (2)	0.0643 (12)	
O7	1.2084 (6)	0.0724 (2)	0.3535 (2)	0.0534 (11)	
H5A	1.330 (8)	0.032 (4)	0.431 (3)	0.043 (15)*	

### Atomic displacement parameters (Å^2^)

**Table:**

	*U*^11^	*U*^22^	*U*^33^	*U*^12^	*U*^13^	*U*^23^
C1	0.050 (3)	0.052 (3)	0.025 (2)	0.002 (3)	0.002 (2)	−0.005 (2)
C2	0.053 (3)	0.039 (3)	0.037 (3)	−0.001 (3)	−0.001 (3)	0.005 (2)
C3	0.042 (3)	0.037 (3)	0.035 (3)	−0.002 (2)	0.000 (2)	−0.002 (2)
C4	0.036 (3)	0.039 (3)	0.034 (3)	0.002 (2)	−0.005 (2)	−0.003 (2)
C5	0.050 (3)	0.040 (3)	0.044 (3)	0.000 (3)	−0.004 (3)	0.002 (2)
C6	0.054 (3)	0.051 (3)	0.043 (3)	0.002 (3)	−0.010 (3)	−0.018 (3)
C7	0.041 (3)	0.030 (2)	0.030 (3)	−0.002 (2)	−0.004 (2)	0.000 (2)
C8	0.035 (3)	0.039 (3)	0.033 (3)	0.000 (2)	−0.004 (2)	0.002 (2)
C9	0.047 (3)	0.042 (3)	0.038 (3)	−0.013 (3)	0.001 (2)	0.006 (2)
C10	0.042 (3)	0.032 (3)	0.043 (3)	−0.004 (2)	−0.001 (2)	0.002 (2)
C11	0.108 (6)	0.053 (4)	0.054 (4)	0.004 (4)	−0.006 (4)	0.015 (3)
C12	0.059 (4)	0.065 (4)	0.047 (3)	0.008 (3)	0.012 (3)	0.000 (3)
C13	0.048 (3)	0.042 (3)	0.055 (4)	0.004 (3)	0.003 (3)	−0.004 (3)
C14	0.037 (3)	0.039 (3)	0.045 (3)	−0.002 (2)	0.002 (2)	0.004 (2)
C15	0.039 (3)	0.046 (3)	0.049 (3)	0.005 (3)	0.002 (3)	0.008 (3)
C16	0.051 (3)	0.052 (3)	0.054 (4)	0.007 (3)	0.002 (3)	−0.011 (3)
C17	0.055 (4)	0.064 (4)	0.045 (3)	−0.002 (3)	−0.006 (3)	0.004 (3)
C18	0.054 (4)	0.063 (4)	0.049 (4)	−0.006 (3)	−0.001 (3)	−0.004 (3)
C19	0.053 (4)	0.105 (6)	0.049 (4)	−0.014 (4)	−0.013 (3)	0.012 (4)
C20	0.066 (4)	0.082 (5)	0.073 (5)	0.019 (4)	−0.013 (4)	0.021 (4)
C21	0.062 (4)	0.057 (4)	0.074 (5)	0.020 (3)	−0.007 (4)	0.008 (3)
N1	0.070 (4)	0.081 (4)	0.037 (3)	0.018 (3)	−0.002 (3)	−0.004 (3)
N2	0.057 (3)	0.041 (3)	0.051 (3)	−0.010 (2)	0.009 (3)	−0.007 (2)
N3	0.062 (3)	0.033 (2)	0.036 (2)	0.002 (2)	−0.002 (2)	0.0105 (19)
N4	0.040 (2)	0.042 (2)	0.035 (2)	0.003 (2)	0.0034 (19)	0.0010 (19)
N5	0.051 (3)	0.048 (3)	0.054 (3)	0.008 (2)	−0.008 (2)	0.008 (3)
O1	0.122 (5)	0.099 (4)	0.045 (3)	0.009 (4)	−0.011 (3)	−0.026 (3)
O2	0.158 (6)	0.094 (4)	0.046 (3)	−0.004 (4)	0.003 (4)	0.015 (3)
O3	0.066 (3)	0.065 (3)	0.058 (3)	−0.014 (2)	−0.012 (2)	−0.016 (2)
O4	0.099 (4)	0.040 (2)	0.068 (3)	−0.005 (2)	0.012 (3)	−0.001 (2)
O5	0.076 (3)	0.044 (2)	0.054 (2)	0.010 (2)	−0.002 (2)	0.006 (2)
O6	0.086 (3)	0.063 (3)	0.044 (3)	−0.011 (3)	0.009 (2)	0.013 (2)
O7	0.060 (3)	0.055 (3)	0.045 (2)	0.017 (2)	0.003 (2)	0.007 (2)

### Geometric parameters (Å, º)

**Table:**

C1—C6	1.362 (8)	C12—H12A	0.9600
C1—C2	1.372 (7)	C12—H12B	0.9600
C1—N1	1.448 (7)	C12—H12C	0.9600
C2—C3	1.372 (7)	C13—N5	1.316 (7)
C2—H2	0.9300	C13—C14	1.374 (8)
C3—C4	1.406 (7)	C13—H13	0.9300
C3—N2	1.476 (7)	C14—C18	1.415 (8)
C4—C5	1.404 (7)	C14—C15	1.418 (7)
C4—C7	1.460 (7)	C15—C16	1.402 (8)
C5—C6	1.385 (8)	C15—C21	1.408 (8)
C5—H5	0.9300	C16—C17	1.342 (8)
C6—H6	0.9300	C16—H16	0.9300
C7—C8	1.391 (7)	C17—N5	1.346 (8)
C7—C10	1.425 (7)	C17—H17	0.9300
C8—O7	1.267 (6)	C18—C19	1.357 (10)
C8—N4	1.381 (6)	C18—H18	0.9300
C9—O6	1.235 (6)	C19—C20	1.379 (10)
C9—N3	1.369 (7)	C19—H19	0.9300
C9—N4	1.373 (7)	C20—C21	1.346 (10)
C10—O5	1.236 (6)	C20—H20	0.9300
C10—N3	1.404 (7)	C21—H21	0.9300
C11—N3	1.452 (7)	N1—O2	1.211 (7)
C11—H11A	0.9600	N1—O1	1.228 (8)
C11—H11B	0.9600	N2—O3	1.209 (6)
C11—H11C	0.9600	N2—O4	1.234 (6)
C12—N4	1.455 (7)	N5—H5A	0.93 (6)
			
C6—C1—C2	120.6 (5)	N5—C13—C14	120.4 (5)
C6—C1—N1	121.1 (5)	N5—C13—H13	119.8
C2—C1—N1	118.3 (5)	C14—C13—H13	119.8
C3—C2—C1	118.9 (5)	C13—C14—C18	122.8 (6)
C3—C2—H2	120.6	C13—C14—C15	118.3 (5)
C1—C2—H2	120.6	C18—C14—C15	118.9 (5)
C2—C3—C4	123.8 (5)	C16—C15—C21	122.4 (6)
C2—C3—N2	115.1 (5)	C16—C15—C14	118.5 (5)
C4—C3—N2	120.9 (5)	C21—C15—C14	119.1 (6)
C5—C4—C3	114.5 (5)	C17—C16—C15	119.5 (6)
C5—C4—C7	121.0 (5)	C17—C16—H16	120.2
C3—C4—C7	124.4 (4)	C15—C16—H16	120.2
C6—C5—C4	122.3 (5)	C16—C17—N5	120.5 (6)
C6—C5—H5	118.9	C16—C17—H17	119.8
C4—C5—H5	118.9	N5—C17—H17	119.8
C1—C6—C5	120.0 (5)	C19—C18—C14	119.5 (6)
C1—C6—H6	120.0	C19—C18—H18	120.3
C5—C6—H6	120.0	C14—C18—H18	120.3
C8—C7—C10	120.1 (4)	C18—C19—C20	121.2 (6)
C8—C7—C4	119.6 (4)	C18—C19—H19	119.4
C10—C7—C4	120.0 (4)	C20—C19—H19	119.4
O7—C8—N4	116.1 (4)	C21—C20—C19	121.5 (7)
O7—C8—C7	124.1 (4)	C21—C20—H20	119.3
N4—C8—C7	119.8 (4)	C19—C20—H20	119.3
O6—C9—N3	121.8 (5)	C20—C21—C15	119.9 (7)
O6—C9—N4	120.8 (5)	C20—C21—H21	120.1
N3—C9—N4	117.5 (4)	C15—C21—H21	120.1
O5—C10—N3	118.4 (5)	O2—N1—O1	123.3 (6)
O5—C10—C7	125.4 (5)	O2—N1—C1	119.5 (6)
N3—C10—C7	116.1 (5)	O1—N1—C1	117.2 (6)
N3—C11—H11A	109.5	O3—N2—O4	124.8 (5)
N3—C11—H11B	109.5	O3—N2—C3	118.9 (5)
H11A—C11—H11B	109.5	O4—N2—C3	116.2 (5)
N3—C11—H11C	109.5	C9—N3—C10	124.1 (4)
H11A—C11—H11C	109.5	C9—N3—C11	117.9 (5)
H11B—C11—H11C	109.5	C10—N3—C11	118.0 (5)
N4—C12—H12A	109.5	C9—N4—C8	122.3 (4)
N4—C12—H12B	109.5	C9—N4—C12	119.4 (4)
H12A—C12—H12B	109.5	C8—N4—C12	118.2 (4)
N4—C12—H12C	109.5	C13—N5—C17	122.8 (5)
H12A—C12—H12C	109.5	C13—N5—H5A	117 (3)
H12B—C12—H12C	109.5	C17—N5—H5A	121 (3)
			
C6—C1—C2—C3	−0.1 (9)	C13—C14—C18—C19	178.2 (6)
N1—C1—C2—C3	179.7 (5)	C15—C14—C18—C19	−1.8 (9)
C1—C2—C3—C4	1.9 (9)	C14—C18—C19—C20	2.1 (10)
C1—C2—C3—N2	−172.9 (5)	C18—C19—C20—C21	−0.8 (12)
C2—C3—C4—C5	−2.1 (8)	C19—C20—C21—C15	−0.8 (11)
N2—C3—C4—C5	172.5 (5)	C16—C15—C21—C20	−180.0 (7)
C2—C3—C4—C7	173.1 (5)	C14—C15—C21—C20	0.9 (9)
N2—C3—C4—C7	−12.4 (8)	C6—C1—N1—O2	−177.3 (7)
C3—C4—C5—C6	0.5 (8)	C2—C1—N1—O2	2.9 (10)
C7—C4—C5—C6	−174.8 (6)	C6—C1—N1—O1	1.3 (9)
C2—C1—C6—C5	−1.4 (9)	C2—C1—N1—O1	−178.4 (6)
N1—C1—C6—C5	178.9 (5)	C2—C3—N2—O3	131.3 (5)
C4—C5—C6—C1	1.2 (9)	C4—C3—N2—O3	−43.7 (8)
C5—C4—C7—C8	132.9 (5)	C2—C3—N2—O4	−44.6 (7)
C3—C4—C7—C8	−41.9 (8)	C4—C3—N2—O4	140.4 (5)
C5—C4—C7—C10	−41.3 (7)	O6—C9—N3—C10	−177.7 (5)
C3—C4—C7—C10	143.8 (5)	N4—C9—N3—C10	3.2 (8)
C10—C7—C8—O7	177.1 (5)	O6—C9—N3—C11	0.2 (9)
C4—C7—C8—O7	2.9 (8)	N4—C9—N3—C11	−179.0 (6)
C10—C7—C8—N4	−2.6 (7)	O5—C10—N3—C9	177.3 (5)
C4—C7—C8—N4	−176.8 (5)	C7—C10—N3—C9	−4.4 (8)
C8—C7—C10—O5	−177.8 (5)	O5—C10—N3—C11	−0.5 (8)
C4—C7—C10—O5	−3.6 (8)	C7—C10—N3—C11	177.7 (5)
C8—C7—C10—N3	4.0 (7)	O6—C9—N4—C8	179.4 (5)
C4—C7—C10—N3	178.2 (5)	N3—C9—N4—C8	−1.4 (8)
N5—C13—C14—C18	179.4 (6)	O6—C9—N4—C12	4.0 (8)
N5—C13—C14—C15	−0.6 (8)	N3—C9—N4—C12	−176.8 (5)
C13—C14—C15—C16	1.2 (8)	O7—C8—N4—C9	−178.5 (5)
C18—C14—C15—C16	−178.8 (5)	C7—C8—N4—C9	1.2 (7)
C13—C14—C15—C21	−179.7 (5)	O7—C8—N4—C12	−3.1 (7)
C18—C14—C15—C21	0.4 (8)	C7—C8—N4—C12	176.7 (5)
C21—C15—C16—C17	179.9 (6)	C14—C13—N5—C17	−0.3 (9)
C14—C15—C16—C17	−0.9 (9)	C16—C17—N5—C13	0.6 (9)
C15—C16—C17—N5	0.1 (9)		

### Hydrogen-bond geometry (Å, º)

**Table:**

*D*—H···*A*	*D*—H	H···*A*	*D*···*A*	*D*—H···*A*
N5—H5*A*···O7	0.93 (6)	1.74 (6)	2.592 (6)	150 (5)
C13—H13···O5^i^	0.93	2.40	3.260 (7)	153
C16—H16···O6^ii^	0.93	2.33	3.187 (7)	154
C17—H17···O2^iii^	0.93	2.61	3.424 (8)	146

Symmetry codes: (i) *x*+1/2, −*y*+1/2, −*z*+1; (ii) −*x*+3, *y*−1/2, −*z*+1/2; (iii) −*x*+5/2, −*y*, *z*−1/2.
